# Reproducibility of Scleral Spur Identification and Angle Measurements Using Fourier Domain Anterior Segment Optical Coherence Tomography

**DOI:** 10.1155/2012/487309

**Published:** 2012-11-01

**Authors:** Ricardo J. Cumba, Sunita Radhakrishnan, Nicholas P. Bell, Kundandeep S. Nagi, Alice Z. Chuang, Shan C. Lin, Kimberly A. Mankiewicz, Robert M. Feldman

**Affiliations:** ^1^Ruiz Department of Ophthalmology and Visual Science, The University of Texas Medical School at Houston, 6431 Fannin Street, MSB 7.024, Houston, TX 77030, USA; ^2^Ophthalmology Department, Medical School, University of Puerto Rico, Medical Sciences Campus, P.O. Box 365067, San Juan, PR 00936, USA; ^3^Glaucoma Center of San Francisco, 55 Stevenson Street, San Francisco, CA 94105, USA; ^4^Department of Ophthalmology, University of California, San Francisco, 10 Koret Way, Room K-300, P.O. Box 0730, San Francisco, CA 94143, USA; ^5^Robert Cizik Eye Clinic, 6400 Fannin Street, Suite 1800, Houston, TX 77030, USA; ^6^Department of Ophthalmology, The University of Texas Health Science Center at San Antonio, 7703 Floyd Curl Drive, Mail Code 6230, San Antonio, TX 78229, USA

## Abstract

*Purpose*. To evaluate intraobserver and interobserver agreement in locating the scleral spur landmark (SSL) and anterior chamber angle measurements obtained using Fourier Domain Anterior Segment Optical Coherence Tomography (ASOCT) images. *Methods*. Two independent, masked observers (SR and AZC) identified SSLs on ASOCT images from 31 eyes with open and nonopen angles. A third independent reader, NPB, adjudicated SSL placement if identifications differed by more than 80 **μ**m. Nine months later, SR reidentified SSLs. Intraobserver and interobserver agreement in SSL placement, trabecular-iris space area (TISA750), and angle opening distance (AOD750) were calculated. *Results*. In 84% of quadrants, SR's SSL placements during 2 sessions were within 80 **μ**m in both the *X*- and *Y*-axes, and in 77% of quadrants, SR and AZC were within 80 **μ**m in both axes. In adjudicated images, 90% of all quadrants were within 80 **μ**m, 88% in nonopen-angle eyes, and 92% in open-angle eyes. The intraobserver and interobserver correlation coefficients (with and without adjudication) were above 0.9 for TISA750 and AOD750 for all quadrants. *Conclusions*. Reproducible identification of the SSL from images obtained with FD-ASOCT is possible. The ability to identify the SSL allows reproducible measurement of the anterior chamber angle using TISA750 and AOD750.

## 1. Introduction

Visualization of the anterior chamber angle and an assessment of the angle width are essential for the diagnosis and management of primary angle closure (PAC). Gonioscopy can be used to visualize the angle structures qualitatively; however, quantification of measurements is unreliable [1], since results rely on the physician's subjective assessment [2]. Despite these limitations, the clinical utility of gonioscopy in caring for glaucoma patients has been well documented and has been recommended to be a part of the evaluation of every glaucoma patient [3]. In recent years, several instruments, including anterior segment optical coherence tomography (ASOCT), have been designed to rapidly image and measure the anterior chamber angle [2, 4–9].

Trabecular-iris space area (TISA) and angle opening distance (AOD) are 2 measurements that are commonly used to quantify the anterior chamber angle [10, 11]. These measurements depend upon reliable and reproducible identification of a landmark, which is typically identified as the scleral spur. The actual structure being identified as a landmark is generally unimportant, only that it is reproducible so that changes in anatomy that would occur after lens extraction or peripheral iridotomy can be quantitated. Current technological limitations require that the location of the landmark be determined manually, introducing measurement error. It has been reported that inconsistency in scleral spur identification accounts for 50% of TISA variability [12]. A reproducible method for scleral spur placement would be useful for measuring treatment effect in PAC.

Several previous studies using ASOCT to measure the anterior chamber angle have shown that interobserver and intervisit measurements of the nasal and temporal quadrants are more reproducible than measurements of the superior and inferior angles [7, 13, 14]. Furthermore, measurements of eyes with narrow or closed angles are less reproducible than those obtained in eyes with open angles [12]. 

The Casia SS-1000 Fourier Domain- (FD-) ASOCT (Tomey Corporation, Nagoya, Japan) is a newly developed ASOCT, which uses Fourier domain swept-source technology resulting in a substantial improvement in scan speed (30,000 A-scans per second) when compared to the Visante ASOCT (2,000 A-scans per second; Carl Zeiss Meditec, Inc., Dublin, CA). There are other FD-ASOCT devices that can image the anterior chamber angle; however, because of its scan speed and swept source design, the Casia SS-1000 can image the anterior chamber angle in high resolution and in a wavelength that allows visualization of the angle recess. All current multipurpose commercially available FD-ASOCTs capable of measuring the anterior chamber angle use wavelengths which do not allow consistent clear visualization of the angle recess. 

Liu et al. showed that intervisit-intraobserver and intravisit-interobserver reproducibility were excellent in 30 normal eyes using the Casia SS-1000. The factors affecting the variability of TISA750 were mean TISA750, inferior and superior quadrants, while the variability in AOD750 was correlated with mean AOD750 as well as mean and variability of iris thickness [15]. Fukuda et al. attempted to compare the reproducibility between Casia SS-1000 with the Scheimpflug analyzer in both open- and nonopen-angle eyes [16]; however, they compared anterior chamber volume and did not focus on angle measurements, TISA750 and AOD750. To the best of our knowledge, there is no study evaluating whether the high-resolution images from the swept source Casia SS-1000 translate into improved reproducibility of identifying the scleral spur when performing angle measurements in nonopen-angle eyes. 

The primary aim of this study is to evaluate intraobserver and interobserver agreement in locating the scleral spur landmark (SSL) in both open- and nonopen-angle eyes and the reproducibility of anterior chamber angle measurements (AOD and TISA) from ASOCT images obtained using the swept-source FD-ASOCT (Casia SS-1000). 

## 2. Methods

This prospective selected cohort study was conducted at the Robert Cizik Eye Clinic of the Ruiz Department of Ophthalmology and Visual Science at The University of Texas Medical School at Houston (part of UTHealth), Houston, TX. Institutional Review Board approval was obtained from The University of Texas Health Science Center Committee for the Protection of Human Subjects, and the University of California at San Francisco determined that IRB approval was not needed because only deidentified data was being used by those investigators. All research adhered to the tenets of the Declaration of Helsinki and was HIPAA compliant.

### 2.1. Participants

Participants 18 years of age or older were recruited from patients and staff at the Robert Cizik Eye Clinic. Informed consent was obtained from the participants after explanation of the nature and possible consequences of the study. After obtaining informed consent, demographic data were recorded, and participants underwent slit lamp examination and intraocular pressure (IOP) measurement, followed by gonioscopy examination performed by one of the glaucoma specialists (RMF or NPB) using a Posner goniolens without compression under minimum ambient lighting conditions. Eyes with any previous laser or intraocular surgery or any anterior segment abnormality that affected the angle or its measurements (i.e., significant corneal opacity) were excluded. Gonioscopy was used to stratify the angle anatomy into 1 of 2 groups: open angle (Shaffer score 3-4 for all 4 quadrants) and nonopen angle (Shaffer score 0-1 in at least one quadrant). Eyes having a Shaffer score of 2 were excluded to enhance separation between the groups. Thirty-one participants met eligibility criteria. When both eyes of the participant were eligible, one eye was randomly selected.

### 2.2. ASOCT Imaging

The Casia SS-1000 FD-ASOCT, a swept-source FD-ASOCT, uses 1,310 nm wavelength light with scan speed of 30,000 A-scans per second to image the anterior chamber, including the angle recess. Images can be obtained in high-resolution 2D mode (2048 A-scans each, 1 pixel = 7.9 *μ*m × 10.0 *μ*m) with both horizontal and vertical plane scans simultaneously in 0.2 seconds. All radial scans taken are 16 mm in length and 6 mm in depth.

### 2.3. Acquisition of ASOCT Images

All participants had their angles imaged in a dark room by 2 operators. One opened the participant's eyelids while the other operated the ASOCT. For the operator opening the participant's eyelids, both index fingers were placed at the eyelid margins and the eyelids separated to allow visualization of the superior and inferior limbus. Pressure from the index fingers was directed to the superior and inferior orbital rims to avoid pressure on the globe. Participants were instructed to focus on the internal fixation light. After adjusting the participant's position, eyes were scanned in 2D mode using the autoalignment function.

### 2.4. Analysis of ASOCT Images

#### 2.4.1. Image Analysis Software

The limited capabilities of the Casia SS-1000 FD-ASOCT built-in software prompted the development of customized software, Anterior Chamber Angle and Interpretation (ACAI), which is specifically designed to measure the anterior chamber angle quantitatively from ASOCT images and requires only manual placement of the SSL. With the ACAI software, unlike the built-in Casia software, the images can be edited and saved with edge detection as necessary for manual resolution of inaccurate interface determination (See Supplementary Figure 1 in Supplementary Material available online at doi:10.1155/2012/487309). 

#### 2.4.2. Criteria for Identifying SSL

The criteria for identifying the SSL were as follows.

**Figure 1:**
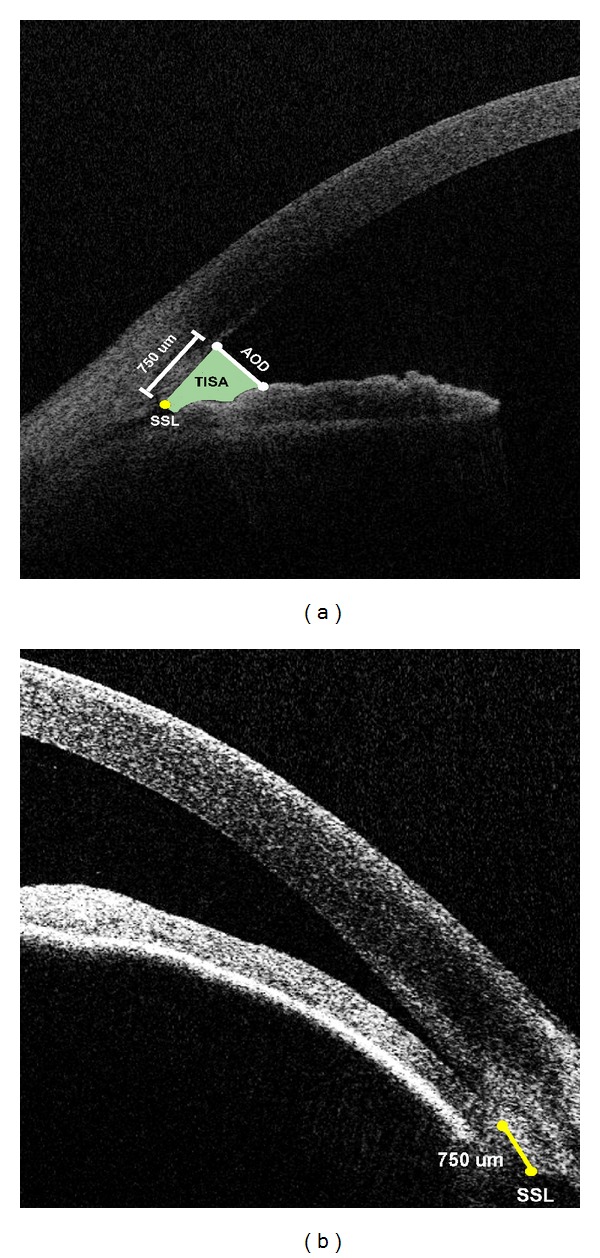
Anterior chamber angle measurements and scleral spur landmark (SSL). Illustrations are angle opening distance (AOD) and trabecular-iris space area (TISA) for an (a) open angle and (b) nonopen angle. AOD is defined as the length of a line drawn perpendicular to the cornea anterior to the SSL and ending on the anterior surface of the iris. TISA is the area bordered centrally by the AOD line, anteriorly by the posterior corneoscleral-aqueous interface, and posteriorly by the anterior surface of the iris. The peripheral border is a line segment starting at the SSL perpendicular to the corneoscleral-aqueous interface and ending on the anterior surface of the iris.

1. The point where there was a change in curvature in the corneoscleral-aqueous interface, often appearing as an inward protrusion of the sclera (Figure 1(a)) [9].
2. If the SSL based on criterion 1 was not clearly visible, the observers were instructed to mark the most posterior end of the trabecular meshwork on the posterior corneoscleral-aqueous interface (Figure 1(b)) in a manner similar to the methods of Usui et al. [17].

While the SSL placement in this study resembles and is in proximity to the true scleral spur, the SSL was meant to be an independent landmark determined by the preset criteria above and not necessarily the true position of the scleral spur. 

#### 2.4.3. Training and Reading Procedure

A nonexpert observer (AZC) with limited knowledge of ocular anatomy was trained to identify the SSL by a glaucoma specialist (RMF) at the Robert Cizik Eye Clinic using a training set of 10 images. For the study, all images were read by 2 observers, who identified both SSLs on each image independently. One was a glaucoma specialist (SR) with extensive experience analyzing ASOCT images [11, 14] and the other was the trained nonexpert (AZC). The images were reread 9 months later by SR for assessing intraobserver agreement. These images were also graded for visibility of the SSL based on criterion 1 (0 = not  visible, 1 = visible). When the observers reviewed the images, they recorded the pixel coordinates of each SSL. The origin of the pixel coordinates was located at the center of the image and rescaled into micrometer (*μ*m) units, 1 pixel = 7.9 *μ*m × 10.0 *μ*m. 

### 2.5. Calculation of Angle Parameters

Using these SSL locations, AOD750 and TISA750 were calculated. AOD750 is defined as the length of a line drawn perpendicular to the cornea starting 750 *μ*m anterior to the SSL and ending on the anterior surface of the iris. TISA750 is the area bordered centrally by the AOD750 line, anteriorly by the posterior corneoscleral-aqueous interface, and posteriorly by the anterior surface of the iris. The peripheral border is a line segment starting at the SSL perpendicular to the corneoscleral-aqueous interface and ending on the anterior surface of the iris (Figure 1(a)).

### 2.6. Adjudication and Threshold

When the SSL locations differed by 80 *μ*m (approximately 20% of the length of TM [17]) or more in either length (*X*-axis) or in depth (*Y*-axis) by the 2 observers, the images were sent for adjudication by a third masked observer (NPB, a glaucoma specialist). Since the precision of the SSL location has an impact on the angle measurements, TISA750 and AOD750, the predetermined threshold, 80 *μ*m (0.08 mm), for adjudication was determined by the following facts.

**Figure 2:**
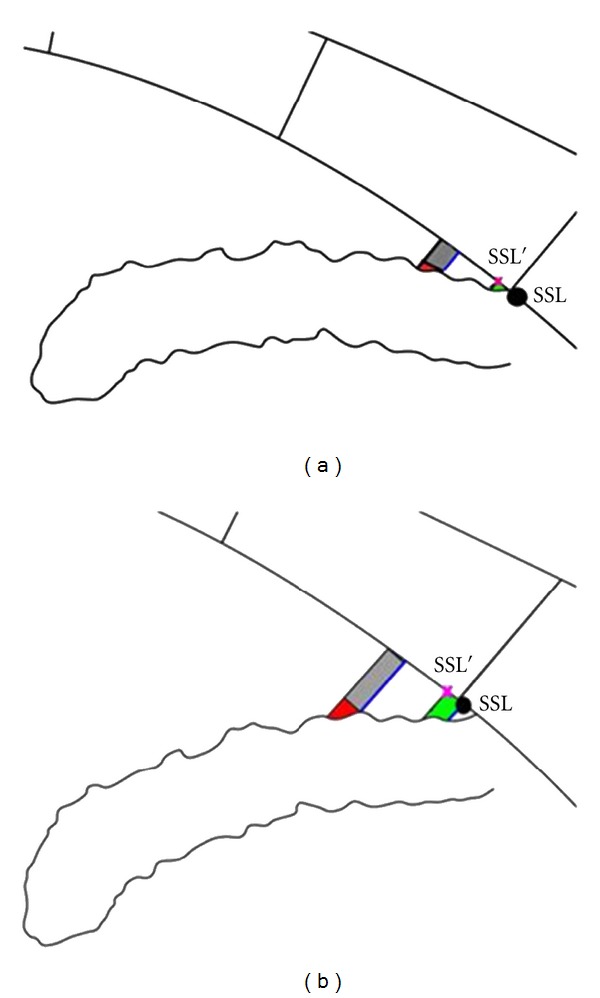
Effect of scleral spur landmark (SSL) location on TISA750 and AOD750. Illustrations show the impact of SSL location on trabecular-iris space area at 750 *μ*m (TISA750) and angle opening distance at 750 *μ*m (AOD750) calculations for nonopen-angle eyes (a) and open-angle eyes (b). If the SSL location is moved by *h* to SSL′ location, TISA750, now based on SSL′, gains the grey area and red area and loses the green area. Both the green area and red area are small relative to the grey area, especially in nonopen-angle eyes (a). In addition, the difference between the green and red areas are negligible (gain red and lose green). Thus, when SSL is moved to SSL′, TISA750 is changed by *h* × AOD750 based on SSL (longer blue line). The SSL location's impact on AOD750 is dependent on angle width and iris shape (the length of the anterior border of red area). The AOD750 changes are smaller if the angle is nonopen and the iris is straightener.

1. TISA750 is a roughly trapezoidal area with the AOD750 as the long side (Figure 2).
2. If the SSL placements differ by *h* mm (distance between SSL and SSL′ in Figure 2), the TISA750 is approximately different by *h* × AOD750 mm^2^ in narrow or closed angles (Figure 2(a) grey area) because the TISA750 based on SSL′ including the grey and red areas but not the green area. Both the green and red areas are small and approximately the same size (Figure 2) [15].
3. The mean AOD750 in narrow angles was described in Console et al. [12] and estimated to be 0.161 mm.
4. See et al. reported that after laser peripheral iridotomy, TISA750 was increased 0.05 mm^2^ with 95% confidence intervals of 0.03 and 0.07 mm^2^; AOD750 was not reported [18].

Thus, in order to obtain a measurement error less than 0.03 mm^2^ in TISA750 (lower confidence limit for changing TISA750 after peripheral iridotomy) in narrow angles [18], the threshold, *h*, should be less than or equal to 0.09 mm ( = [0.03/0.161]/1.96). For convenience and consistency, the 0.08 mm threshold was selected because it is less than 0.09 mm and corresponds to 10 pixels in the *X*-axis, with the same threshold for the *Y*-axis, even though TISA750 is less affected by errors in the *Y*-axis.

### 2.7. Statistical Analysis

Demographics were summarized by mean and standard deviation (SD) for continuous variables or by frequency (%) for discrete variables. Differences between measurements (sessions or observers) were calculated for both the *X*- and *Y*-axes. Intraobserver agreement was calculated for the glaucoma specialist (SR) identifying the SSL in 2 sessions. Interobserver agreement was calculated using 2 observers (nonexpert and glaucoma specialist SR) for all measurements. Interobserver agreement was recalculated using the final pair of locations identified by 2 glaucoma specialists (SR and NPB), when the initial 2 observations were greater than 80 *μ*m apart. The percentages of these differences within 80 *μ*m were computed for intraobserver, interobserver without adjudication, and interobserver with adjudication. In addition, intraobserver and interobserver agreement were evaluated using the mean (bias) and standard deviation of these differences as well as 95% limits of agreement (bias ± 1.96 × SD). 

The sample size calculation was based on the precision, standard error of estimated percentage that 2 observers identified the SSL within 80 *μ*m, and the standard error of the agreement limits on the Bland-Altman plot. In addition, the SSL *X*- and *Y*-coordinates will vary depending on where on the Cartesian plane the imaged eye falls. Because the eye is not perfectly round and is often imaged in a slightly tilted position, especially in the horizontal cross-section, the SSL locations within an eye are not correlated. A sample size of 124 angles from 31 eyes would provide the standard error of estimated percentage less than 4.5% ($=\sqrt{\left(0.5\times 0.5\right)/124}$). In addition, based on Console et al. [12], the standard deviation of difference, *σ*_d_, is approximately 2.6 pixels (or 70.2 *μ*m), and the sample size of 124 angles would provide the standard error of the agreement limits to be 11 *μ*m ($=\sqrt{3{(70.2}^{2}/124})$).

The averages and differences between pairs of TISA750 obtained by the glaucoma specialist (SR) in 2 different sessions as well as 2 observers with and without adjudication were calculated. The mean (±SD) differences were computed. The agreement of TISA750 was also evaluated with Bland-Altman plots. Similarly, the AOD750 agreement was evaluated using the same statistical methods. In addition, a random intercept model was used to calculate the intraclass correlation coefficient (ICC) as a measure of intraobserver and interobserver reproducibilities for TISA750 and AOD750. An ICC ≤ 0.4 was defined as poor reproducibility, between 0.4 and 0.75 was defined as fair to good reproducibility, and ≥0.75 was defined as excellent reproducibility [14]. 

The observed difference (both intra- and interobserver) as a proportion of the standard deviation for TISA750 and AOD750 were computed. There are 2 standard deviations, one is the standard deviation of paired observed differences (within angle same angle, multiple readings, denoted as SD_d_) and the other is the total variability of TISA750 or AOD750 including within and total variation (denoted as SD_TISA_ and SD_AOD_). 

Due to the potential correlations between the angle measurements obtained for an individual eye, the secondary analyses conducted used a mixed-effect model for comparing the means between groups and between horizontal and vertical scans and used a generalized estimating equation (GEE) with logit link comparing the percentages. Finally, the impact of SSL location on TISA750 and AOD750 was evaluated by estimating the changes in TISA750 and in AOD750 when the SSL distance (without adjudication) increased by 10 *μ*m using mixed-effect models. 

All statistical analyses were performed using SAS for Windows v9.2 (SAS, Inc., Cary, NC). *P* < 0.05 was considered statistically significant for all comparisons.

## 3. Results

A total of 31 eyes of 31 consecutive qualifying subjects, classified as open angle (12 eyes) and nonopen angle (19 eyes), were included in this study. Fourteen (45.2%) of the 31 study eyes were right eyes. There were 18 women (58.1%), and the mean age was 54.4 ± 15.6 years (range 26–78 years). The study included 18 White (58.1%), 5 Hispanic (16.1%), 4 Black (12.9%), and 4 Asian (12.9%) participants. All images were included in the statistical analysis. 

### 3.1. Scleral Spur Landmark Visibility

SSL could not be identified by criterion 1 in 17 of 124 angles (14%). Of those 9 were in the inferior, 7 superior, and 1 temporal quadrants. The SSLs in these angles were identified using criteria 2 (the posterior end of trabecular meshwork on the posterior corneoscleral-aqueous interface). Using criteria 1 and 2 sequentially, there were no images in which the SSL could not be identified. 

### 3.2. Intraobserver Agreement of SSL Placement

Differences between SSL locations for both the *X*- and *Y*-axes were within 80 *μ*m in 104 of 124 quadrants (84%) evaluated by the glaucoma specialist (SR) in 2 separate sessions, 9 months apart (88% for *X*-axis and 94% for *Y*-axis). Agreement in open angles was lowest in both the superior and inferior quadrants (67%). Agreement in nonopen angles was lowest in the nasal quadrant (79%). Agreement within 80 *μ*m in the temporal quadrant was 89% for nonopen eyes and 100% for open-angle eyes. The mean (±SD) difference in identified locations was −6.2 (±52.5) with 95% limits of agreement from −109.1 to +96.7 *μ*m and −4.6 (±41.3) *μ*m with 95% limits of agreement from −85.5 to +76.3 for the *X*-axis and *Y*-axis, respectively. 

### 3.3. Interobserver Agreement of SSL Placement

The number (%) of SSLs identified within 80 *μ*m in *X*- and *Y*-axes by the 2 observers with or without adjudication is reported in Table 1. In 77% of all quadrants, the 2 independent observers were within 80 *μ*m in both the *X*- and *Y*-axes. There were 28 (23%) angles that met criteria for adjudication. The adjudicator value replaced the nonexpert data for further analysis. After adjudication, 90% of all quadrants were within the 80 *μ*m threshold: 92% in nonopen-angle eyes and 88% in the open-angle eyes. When analyzed by quadrant, 29% of superior and inferior quadrants required adjudicator review, while 13% and 19% of temporal and nasal quadrants, respectively, required review. 

**Figure 3:**
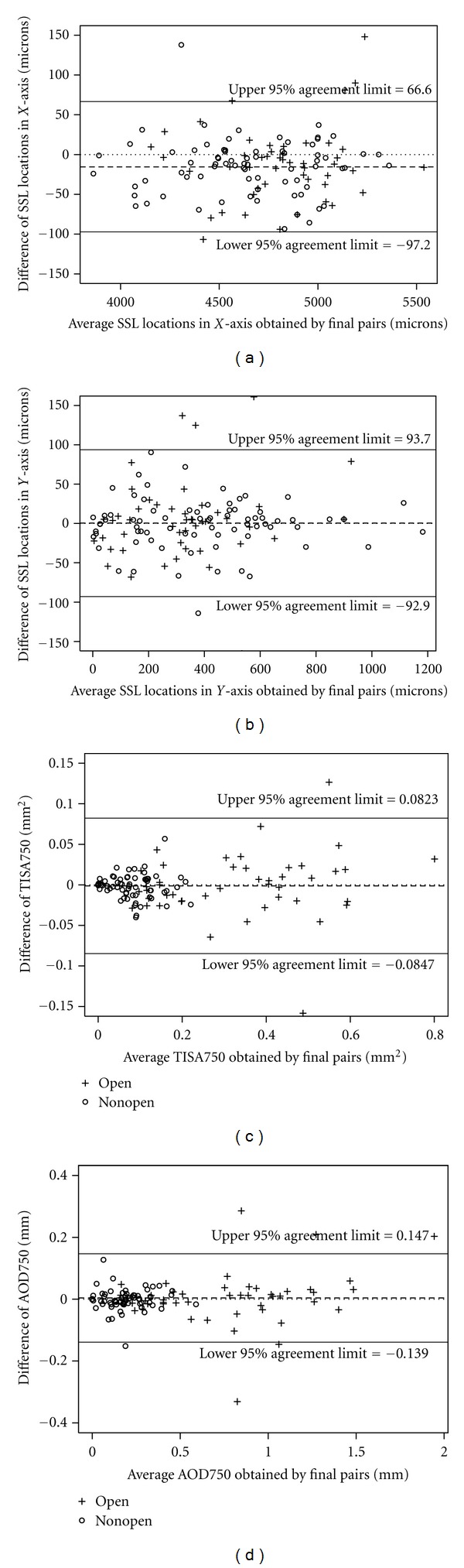
Bland-Altman agreement plots for adjudicated pairs for (a) SSL location in *X*-axis, (b) SSL location in *Y*-axis, (c) TISA750, and (d) AOD750.

The mean (±SD) differences in SSL placement with and without adjudication are shown in Table 2. The mean difference of the final pairs of SSLs in the *X*-axis was −15.5 *μ*m, with 95% limits of agreement being −97.2 and +66.6 *μ*m (Figure 3(a)), while the mean difference in the *Y*-axis was 0.4 *μ*m with 95% limits of agreement being −92.9 and +93.7 *μ*m (Figure 3(b)).

The mean differences (bias) in both the *X*- and *Y*-axes were similar before and after adjudication (Table 2). Variation in the *X*-axis was reduced by 22% after adjudication (from 53.3 *μ*m between the 2 observers without adjudication to 41.8 *μ*m with adjudication). Similarly, the variation in the *Y*-axis was reduced by 10% after adjudication. These reductions in variation due to the adjudication were mainly observed in nonopen-angle eyes (36% in *X*-axis and 26% in *Y*-axis), while no reductions were seen in open-angle eyes.

### 3.4. Intraobserver Reproducibility for TISA750 and AOD750

Table 3 summarizes the means (±SD) of TISA750 and AOD750 resulting from the SSLs identified by the glaucoma specialist (SR) in 2 sessions. The mean difference in TISA750 was 0.0087 (±0.0493) mm^2^ for open-angle eyes and 0.0037 (±0.0141) mm^2^ for nonopen-angle eyes. Similarly, the mean difference in AOD750 was 0.030 (±0.171) mm for open-angle eyes and 0.008 (±0.034) mm for nonopen-angle eyes. The intraobserver correlation coefficients (ICC) for all angles grouped by angle status as well as quadrants are shown in Table 4. The results indicate that all ICCs are excellent (>0.75). In addition, all ICCs were >0.9, except for the AOD750 at the temporal quadrant in open-angle eyes (=0.81). The observed differences between 2 sessions were less than 10% of standard deviation in all eyes. The subgroup analysis showed the largest observed difference was 17% of standard deviation at superior open angle.

### 3.5. Interobserver Reproducibility for TISA750 and AOD750

The means (±SD) of TISA750 and AOD750 obtained from the SSLs identified by 2 observers without adjudication and with adjudication are summarized in Tables 5 and 6. The interobserver biases and variations in TISA750 and AOD750 were similar with and without adjudicator review. The horizontal (temporal and nasal) scan was the deepest while the vertical (superior and inferior) scan was narrowest (*P* = 0.0041 and 0.0004 for TISA750 and AOD750, resp., using the adjudicated pairs). In addition, the variations of TISA750 and AOD750 obtained by 2 observers in nonopen-angle eyes were approximately 33% (0.0174 versus 0.0530) and 35% (0.034 versus 0.097) of the variations in the open-angle eyes, respectively. The 95% limits of agreement for TISA750 and AOD750 with adjudication were −0.0847 to +0.0823 mm^2^ and −0.139 to +0.147 mm, respectively, for all quadrants. Figures 3(c) and 3(d) show the Bland-Altman agreement plots for TISA750 and AOD750.

The ICC without adjudication (and with adjudication) for all quadrants was 0.98 (0.97) for TISA750 and 0.96 (0.94) and 0.96 (0.97) for the open- and nonopen-angle groups, respectively. For AOD750, the ICC was 0.99 (0.98) for all eyes and 0.97 (0.97) and 0.97 (0.97) for the open- and nonopen-angle groups, respectively (Table 7). Based on the predefined criteria of ICC > 0.75 considered as excellent, the reproducibility for both TISA750 and AOD750 was excellent with and without adjudication. The observed differences between observers (with or without adjudication) were less than 10% of standard deviation in all eyes. The largest observed differences were found in superior and inferior angle of open-angle eyes.

### 3.6. Impact of SSL Location on TISA750 and AOD750

The mean (±SD) distances between 2 SSLs identified by 2 observers were 54.6 (±44.9) *μ*m in open angles and 57.1 (±56.7) *μ*m in nonopen angles. When the SSL distance increases 10 *μ*m, the mean TISA750 increased by 0.0066 (±0.0009) mm^2^ in open-angle eyes (*P* < 0.0001) and 0.0012 (±0.0002) mm^2^ in nonopen-angle eyes (*P* < 0.0001). Similarly, the mean AOD750 increased by 0.013 (±0.002) mm in open-angle eyes (*P* < 0.0001) and 0.002 (±0.000) mm^2^ in nonopen-angle eyes (*P* < 0.0001) as the SSL distance increases 10 *μ*m. 

Of the 76 quadrants in nonopen-angle eyes, TISA750 differed by more than 0.03 mm^2^, the lower confidence interval of the effect of peripheral iridotomy (see Section 2.6) between the 2 observers in 7 quadrants (9.2%). Among these 7 quadrants, 6 quadrants had SSL distances that differed by more than 80 *μ*m. After adjudication, 4 of 6 quadrants were no longer differed in TISA750 by more than 0.03 mm^2^. Twelve quadrants had SSL placement adjudication while their TISA750 were within 0.03 mm^2^.

### 3.7. Post Hoc Analysis of Correlation and Sample Size

Post hoc analysis was conducted to evaluate the correlation within an eye.  Table 8 uses our data to evaluate the correlation among four quadrants within an eye, where *r* is the correlation of coefficient. From this table, |*r*| is less than 0.1 for SSL location-related measurements (agreement or distance), and therefore uncorrelated, while the correlation of TISA within an eye is >0.9 (highly correlated). Thus, at 5% significant level, the sample size in this study had a statistical power of 77% to detect a 20% difference in agreement using GEE models validating our *a priori* assumptions.

## 4. Discussion

The gold standard for visualization of the anterior chamber angle is gonioscopy, a subjective technique. In contrast, ASOCT provides objective assessment of the anterior chamber angle anatomy, and quantitative measurements such as AOD and TISA can be obtained. However, both of these parameters require a fixed reference point to obtain accurate measurements. Reliable automated detection of this reference point has remained elusive; hence, manual identification of the scleral spur is used. However, this introduces another element of variability which is observer dependent. This study demonstrates that a nonexpert observer can be trained to read similarly to the expert. Reproducible identification of the SSL is possible with both intraobserver and interobserver variability within limits that may result in clinically useful measurements of AOD750 and TISA750. 

The method of using 2 masked observers followed by an adjudication if those 2 observers do not reach the same conclusion about the image or data that they are reading is a technique that has been used previously with success in many large, NEI-funded clinical trials [19–22]. For example, in clinical trials where large amounts of data are accumulated and a reliable outcome is of crucial importance, this method has been successful in analyzing optic disc photos and visual fields. However, being that ASOCT is emerging as a more commonly used technique that can provide quantitative information about the angle, its use in large-scale clinical trials to quantify changes before and after procedures that may modify angle anatomy (such as lens extraction or peripheral iridotomy) is inevitable. Here, on a small scale, we have validated using the technician-adjudicator technique to analyze ASOCT images in a clinical trial-like setting. 

In this study, the interobserver reproducibility for identifying the SSL (without adjudication) in all images was good overall. In 77% of quadrants, the 2 observers identified the SSL within 80 *μ*m in both the *X*- and *Y*-axes. Sakata et al. reported that 54 of 132 (41%) images from the Visante ASOCT used for reproducibility evaluation were not adequate to identify the scleral spur. Additionally, they reported that identifying the scleral spur was more difficult in the superior and inferior quadrants, possibly related to eyelid obstruction [9]. This led other investigators to only utilize images in the nasal and temporal quadrants [6, 12, 23]. In our study, the high-resolution images obtained with the Casia SS-1000 FD-ASOCT and the use of 2 operators to minimize lid blockage enabled identification of the SSL in all quadrants and SSLs, based on criterion 1 identification, were visible in 86% of quadrants. SSL placement agreement was better in temporal/nasal images compared to superior/inferior images. The percentage of agreement in all eyes between the 2 observers (without adjudication) was 87% in the temporal quadrant, 81% in the nasal quadrant, and 71% in both inferior and superior quadrants. 

Agreement in placement of the SSLs (without adjudication) was not significantly different (*P* = 0.7116) in the 2 angle anatomy groups: 79% open-angle and 76% nonopen-angle eyes. This is different than the results from Sakata who reported that identification of the scleral spur was more difficult in closed angles [9]. There may be 3 explanations for this: (1) the higher resolution of the Casia SS-1000 FD-ASOCT; (2) the ability to manipulate image contrast with the ACAI software to differentiate 2 different high reflectivity structures that are in contact; (3) definitions of angle anatomy classification may differ between studies. 

Console et al. used temporal/nasal images from the Visante ASOCT to investigate interobserver agreement in scleral spur placement between 2 observers in open and narrow angles. The width of agreement limits for both the nasal and temporal quadrants in the *X*- and *Y*-axes ranged from 261 to 292 *μ*m [12]. In the current study, the width of agreement limits ranged from 160 to 282 *μ*m without adjudication, while the width of agreement limits of the corresponding quadrants ranged from 138 to 178 *μ*m with adjudication. This indicates that the methodology of adjudication used in the current study can reduce interobserver variability by 13%–37%. It may be worth investigating whether this methodology is as effective with images obtained from other devices.

This study was designed to evaluate the potential to use measurements determined from Casia SS-1000 images for clinical trials. The methodology of using an adjudicator to resolve differences between observers has been validated and used in reading centers for other images and devices in many prospective studies [19–22]. With this methodology, reproducibility was excellent within the limits required for angle measurements; 90% of adjudicated angles were within the 80 *μ*m threshold for both the *X*- and *Y*-axes. 

The bias and standard deviation of difference in TISA750 and AOD750 were similar with and without adjudication. However, if one used the closest 2 of 3 measurements as the adjudication result, the SD for all quadrants were reduced from 0.0426 to 0.0304 (29%) for TISA750 and from 0.072 to 0.052 (26%) for AOD750. This reduction in variability may not increase accuracy but if one is looking to determine change over time might be useful.

Eighty microns (a distance approximately 20% of the length of TM [17]) were chosen *a priori* as the acceptable difference between measurements by first determining from the literature the expected change as measured by ASOCT from performing peripheral iridotomy for the angle closure spectrum of disease [18]. Then, the amount of error in SSL placement which would result in that difference was calculated (see Section 2.6.; 0.09 mm^2^ = (0.03/0.161)/1.96). A difference less than the amount of change from a peripheral iridotomy would be acceptable for a study wishing to evaluate whether there was anatomic improvement with treatment and, thus, was felt to be an acceptable minimum detectable difference between images. It was unknown at that time whether such a small difference could be detected reproducibly so the adjudication technique was invoked to reduce interobserver variability.

Identification of the SSL may have clinical implications in that iridotrabecular contact anterior to the SSL can be used to define angle closure. Additionally, quantitative parameters such as the TISA and AOD, which require identification of the SSL as a reference point, may be useful for following patients with clinically narrow angles. While in this paper we have used the wording “scleral spur landmark” to refer to the reference point used to calculate TISA750 and AOD750, choosing the actual anatomical scleral spur is not crucial to calculating angle parameters as long as it can be done consistently. Given that limitation, the actual anatomic structure marked probably has little effect on whether a change can be detected over time. Reproducible identification of the SSL (identified by criteria 1 and 2 in Section 2) enables reproducible quantitative angle measurements as evidenced by the excellent interobserver agreement of TISA750 and AOD750. TISA750 ICC and AOD750 ICC were greater than 0.95 regardless of angle anatomy.

While it is true that there is a correlation for TISA and AOD measurements, the SSLs are not correlated because (1) eyes are not perfectly round and (2) the eye is often imaged in a slightly tilted position, especially in a horizontal cross-section. The vertical tilting is likely caused by the height of the subject. Therefore, the SSL *X*- and *Y*-coordinates in each quadrant will not vary depending on where on the Cartesian plane the imaged quadrant falls. TISA and AOD are not affected by where on this plane it falls because the relative locations of the coordinates are unaffected. Post hoc analysis (Table 8) illustrated this point using our data. With this information, it appears that our sample size calculation statistical methodology and *a priori* assumptions are all appropriate. 

This study has several limitations. First, the study included only eyes without anterior segment abnormalities that would prevent ASOCT imaging (i.e., significant corneal opacity, pterygium, etc.). Thus, the results are only generalizable to this population. Second, while the Casia SS-1000 has the capability of obtaining images in 3D mode in a resolution similar to the Visante ASOCT, this study was limited to images obtained in the higher resolution 2D mode. The excellent reproducibility may not apply to 3D mode images. Third, the adjudicator model used in the current study does not totally mimic the reading center model used in previous randomized clinical trials in that the readers were one expert and one nonexpert (one physician and one layperson), whereas in the typical reading center there are 2 lay readers with a physician adjudicator [19, 21]. Since the purpose of this study was to determine whether measurements could be obtained reproducibly, it was advantageous to compare expert to nonexpert evaluations, which would likely maximize the expected differences. Fourth, the study was also limited by the relatively small sample size and exclusion of Shaffer grade 2 angles, which makes generalizability to grade 2 angles not possible in clinical trials nor in clinical practice. Also in this study, the reproducibility was measured relative to the placement of the SSL on the image and not due to the imaging technique itself. Reproducibility of the imaging technique would require several different operators to take images and then comparison of those images. This is the subject of ongoing research. 

In conclusion, reproducible identification of the SSL from images obtained with the Casia SS-1000 FD-ASOCT is possible. The ability to identify the SSL allows the reproducible measurement of the anterior chamber angle using AOD750 and TISA750. 

## Supplementary Material

Illustrations are Edit and Save Functions in Anterior Chamber Analysis and Interpretation (ACAI) software. After manually identifying the scleral spur landmark (SSL [SS in the figure]), click on the edge detection button (not shown), and the anterior and posterior corneal edges as well as the anterior iris are automatically detected (A, yellow lines). In some eyes, especially nonopen angle eyes, iris surface detection may need manual adjustment (B, green line). Trabecular Iris Surface Area at 750 **μ**m (TISA750) and Angle Opening Distance at 750 **μ**m (AOD750) can be calculated based on the identified SSL and the edited edges (not shown). The edited iris can then be saved to a file (C). To compare TISA750 and AOD750 calculations obtained from the other set of SSL identifications on the same image, the saved iris file would be loaded first (D). After the SSL is identified and the edges automatically detected (by the yellow lines, as in A), the user then clicks on “Get Edit Iris” (E). The iris surface edge is from the previous saved iris file (F). The impact of SSL on TISA750 and AOD750 can then be examined with the same manually edited iris edges.

## Figures and Tables

**Table 1:** Frequency (%) where scleral spur landmark identification was within 80 *μ*m.

	All eyes (*N* = 31) by 2 observers (with adjudicator)	Open-angle eyes (*N* = 12) by 2 observers (with adjudicator)	Nonopen-angle eyes (*N* = 19) by 2 observers (with adjudicator)
*x*-axis			

All quadrants	106 (85%) (118 (95%))	44 (92%) (46 (96%))	62 (82%) (72 (95%))

Horizontal			
Nasal	25 (81%) (29 (94%))	9 (75%) (11 (92%))	16 (84%) (18 (95%))
Temporal	28 (90%) (30 (97%))	12 (100%) (12 (100%))	16 (84%) (18 (95%))
Vertical			
Superior	25 (81%) (29 (94%))	11 (92%) (11 (92%))	14 (74%) (18 (95%))
Inferior	28 (90%) (30 (97%))	12 (100%) (12 (100%))	16 (84%) (18 (95%))

*P* value (H versus V)	1.000 (1.000)	0.3173 (1.000)	0.5271 (1.000)

*y*-axis			

All quadrants	108 (87%) (117 (94%))	41 (85%) (44 (92%))	67 (88%) (73 (96%))

Horizontal			
Nasal	29 (94%) (30 (97%))	11 (92%) (12 (100%))	18 (95%) (18 (95%))
Temporal	28 (90%) (29 (94%))	11 (92%) (11 (92%))	17 (89%) (18 (95%))
Vertical			
Superior	27 (87%) (30 (97%))	10 (83%) (11 (92%))	17 (89%) (19 (100%))
Inferior	24 (77%) (28 (90%))	9 (75%) (10 (83%))	15 (79%) (18 (95%))

*P* value (H versus V)	0.1088 (0.7055)	0.1797 (0.3173)	0.3173 (0.5637)

Both *x*- and *y*-axes			

All quadrants	96 (77%) (112 (90%))	38 (79%) (42 (88%))	58 (76%) (70 (92%))

Horizontal			
Nasal	25 (81%) (29 (94%))	9 (75%) (11 (92%))	16 (84%) (18 (95%))
Temporal	27 (87%) (29 (94%))	11 (92%) (11 (92%))	16 (84%) (18 (95%))
Vertical			
Superior	22 (71%) (28 (90%))	9 (75%) (10 (83%))	13 (68%) (18 (95%))
Inferior	22 (71%) (26 (84%))	9 (75%) (10 (83%))	13 (68%) (16 (84%))

*P* value (H versus V)	0.1025 (0.2482)	0.4795 (0.4795)	0.1336 (0.3173)

**Table 2:** Mean (± standard deviation) differences (*μ*m) in scleral spur landmark placement.

	All eyes (*N* = 31) by 2 readers (with adjudicator) (*μ*m)	Open-angle eyes (*N* = 12) by 2 readers (with adjudicator) (*μ*m)	Nonopen-angle eyes (*N* = 19) by 2 readers (with adjudicator) (*μ*m)
*x*-axis difference			

All quadrants	−16.5* ± 53.3 (−15.5 ± 41.8)	−22.0 ± 41.6 (−11.8 ± 47.0)	−13.0 ± 59.5 (−17.8 ± 38.3)

Horizontal			
Nasal	−25.7 ± 71.9 (−19.7 ± 45.4)	−43.0 ± 52.0 (−30.0 ± 44.1)	−14.8 ± 81.5 (−13.2 ± 46.2)
Temporal	−8.7 ± 41.0 (−13.5 ± 35.2)	−13.0 ± 15.7 (−5.8 ± 33.6)	−6.0 ± 51.2 (−18.3 ± 36.3)
Vertical			
Superior	−20.1 ± 49.7 (−6.8 ± 46.7)	−18.7 ± 45.6 (4.7 ± 59.9)	−21.1 ± 53.3 (−14.1 ± 36.0)
Inferior	−11.4 ± 46.2 (−22.0 ± 39.2)	−13.5 ± 41.4 (−16.0 ± 45.3)	−10.1 ± 50.0 (−25.8 ± 35.6)

*P* value (H versus V)	0.8597 (0.7646)	0.3283 (0.3632)	0.6162 (0.6198)

*y*-axis difference			

All quadrants	1.5* ± 52.8 (0.4 ± 47.6)	−8.0 ± 52.7 (−0.8 ± 59.2)	7.5 ± 52.4 (1.2 ± 39.0)

Horizontal			
Nasal	−2.2 ± 50.5 (2.8 ± 43.7)	−14.6 ± 44.3 (−5.1 ± 33.5)	5.6 ± 53.7 (7.8 ± 49.3)
Temporal	10.3 ± 52.2 (5.5 ± 42.7)	10.9 ± 47.1 (13.7 ± 54.9)	9.9 ± 56.5 (0.4 ± 33.5)
Vertical			
Superior	7.2 ± 45.4 (9.9 ± 45.6)	5.2 ± 51.2 (19.0 ± 64.2)	8.5 ± 42.7 (4.1 ± 29.4)
Inferior	−9.2 ± 62.1 (−16.0 ± 55.3)	−33.4 ± 60.9 (−30.8 ± 71.2)	6.0 ± 59.4 (−7.5 ± 42.1)

*P* value (H versus V)	0.5713 (0.3844)	0.4071 (0.5494)	0.9670 (0.5234)

*A negative mean difference indicates that observer 1 is closer to the center of the image.

**Table 3:** Mean (± standard deviation) of TISA750 (mm^2^) and AOD750 (mm) from both sessions by the glaucoma specialist and their differences.

	Session 1	Session 2	Difference
TISA750 (mm^2^)			

All eyes			

All quadrants	0.1722 ± 0.1774	0.1666 ± 0.1724	0.0056 ± 0.0325
Nasal	0.1772 ± 0.1812	0.1721 ± 0.1763	0.0051 ± 0.0183
Temporal	0.1944 ± 0.2069	0.1880 ± 0.1975	0.0064 ± 0.0273
Superior	0.1550 ± 0.1676	0.1427 ± 0.1521	0.0123 ± 0.0336
Inferior	0.1622 ± 0.1559	0.1634 ± 0.1659	−0.0012 ± 0.0450

Open-angle eyes			

All quadrants	0.3370 ± 0.1775	0.3283 ± 0.1711	0.0087 ± 0.0493
Nasal	0.3373 ± 0.1884	0.3290 ± 0.1847	0.0083 ± 0.0228
Temporal	0.3785 ± 0.2248	0.3644 ± 0.2140	0.0141 ± 0.0394
Superior	0.3226 ± 0.1527	0.3002 ± 0.1262	0.0224 ± 0.0515
Inferior	0.3095 ± 0.1495	0.3197 ± 0.1640	−0.0102 ± 0.0710

Nonopen-angle eyes			

All quadrants	0.0681 ± 0.0590	0.0644 ± 0.0555	0.0037 ± 0.0141
Nasal	0.0760 ± 0.0717	0.0730 ± 0.0647	0.0030 ± 0.0151
Temporal	0.0781 ± 0.0597	0.0766 ± 0.0567	0.0015 ± 0.0152
Superior	0.0491 ± 0.0445	0.0433 ± 0.0431	0.0059 ± 0.0123
Inferior	0.0692 ± 0.0570	0.0647 ± 0.0537	0.0045 ± 0.0143

AOD750 (mm)			

All eyes			

All quadrants	0.400 ± 0.401	0.384 ± 0.375	0.016 ± 0.109
Nasal	0.433 ± 0.419	0.411 ± 0.403	0.022 ± 0.053
Temporal	0.448 ± 0.495	0.412 ± 0.414	0.035 ± 0.182
Superior	0.358 ± 0.349	0.340 ± 0.329	0.018 ± 0.050
Inferior	0.360 ± 0.330	0.371 ± 0.362	−0.011 ± 0.096

Open-angle eyes			

All quadrants	0.768 ± 0.408	0.738 ± 0.367	0.030 ± 0.171
Nasal	0.801 ± 0.446	0.766 ± 0.429	0.035 ± 0.075
Temporal	0.901 ± 0.524	0.808 ± 0.410	0.093 ± 0.287
Superior	0.708 ± 0.309	0.676 ± 0.281	0.032 ± 0.066
Inferior	0.662 ± 0.327	0.702 ± 0.364	−0.040 ± 0.150

Nonopen-angle eyes			

All quadrants	0.167 ± 0.131	0.160 ± 0.123	0.008 ± 0.034
Nasal	0.201 ± 0.154	0.187 ± 0.146	0.015 ± 0.034
Temporal	0.161 ± 0.130	0.162 ± 0.109	−0.001 ± 0.037
Superior	0.138 ± 0.108	0.128 ± 0.099	0.009 ± 0.035
Inferior	0.169 ± 0.129	0.162 ± 0.134	0.008 ± 0.030

**Table 4:** Intraobserver correlation coefficient (ICC) for TISA750 and AOD750.

	TISA750			AOD750		
	ICC	|*μ*_d_|/SD_d_	|*μ*_d_|/SD_TISA_	ICC	|*μ*_d_|/ SD_d_	|*μ*_d_|/SD_AOD_
All eyes						

All quadrants	0.982	0.172	0.032	0.961	0.147	0.043
Nasal	0.994	0.279	0.028	0.990	0.415	0.055
Temporal	0.991	0.234	0.032	0.920	0.192	0.081
Superior	0.976	0.366	0.078	0.988	0.360	0.053
Inferior	0.962	0.027	0.007	0.961	0.115	0.032

Open-angle eyes						

All quadrants	0.960	0.176	0.051	0.902	0.175	0.081
Nasal	0.992	0.364	0.045	0.983	0.467	0.080
Temporal	0.983	0.358	0.065	0.810	0.324	0.220
Superior	0.926	0.435	0.166	0.971	0.485	0.109
Inferior	0.904	0.144	0.068	0.907	0.267	0.123

Nonopen-angle eyes						

All quadrants	0.968	0.262	0.066	0.963	0.235	0.061
Nasal	0.976	0.199	0.044	0.970	0.441	0.098
Temporal	0.967	0.099	0.026	0.955	0.027	0.010
Superior	0.954	0.480	0.137	0.941	0.257	0.093
Inferior	0.965	0.315	0.083	0.974	0.267	0.058

**Table 5:** Mean (± standard deviation) TISA750 (mm^2^) from each observer and differences.

	Observer AZC (with adjudicator) (mm^2^)	Observer SR (session 1) (mm^2^)	Difference (with adjudicator) (mm^2^)
All eyes			

All quadrants	0.1757 ± 0.1855 (0.1710 ± 0.1824)	0.1722 ± 0.1774	**0.0035 ± 0.0358** **(−0.0012 ± 0.0426)**
Nasal	0.1858 ± 0.1904 (0.1782 ± 0.1883)	0.1772 ± 0.1812	0.0087 ± 0.0374 (0.0010 ± 0.0301)
Temporal	0.1931 ± 0.2123 (0.1935 ± 0.2120)	0.1944 ± 0.2069	−0.0013 ± 0.0258 (−0.0009 ± 0.0198)
Superior	0.1519 ± 0.1612 (0.1409 ± 0.1439)	0.1550 ± 0.1676	−0.0031 ± 0.0282 (−0.0141 ± 0.0565)
Inferior	0.1722 ± 0.1811 (0.1714 ± 0.1837)	0.1622 ± 0.1559	0.0100 ± 0.0474 (0.0092 ± 0.0517)

Open-angle eyes			

All quadrants	0.3467 ± 0.1875 (0.3364 ± 0.1899)	0.3370 ± 0.1775	0.0097 ± 0.0530 (−0.0006 ± 0.0666)
Nasal	0.3513 ± 0.2004 (0.3412 ± 0.2036)	0.3373 ± 0.1884	0.0140 ± 0.0594 (0.0039 ± 0.0473)
Temporal	0.3765 ± 0.2383 (0.3801 ± 0.2339)	0.3785 ± 0.2248	−0.0021 ± 0.0300 (0.0015 ± 0.0230)
Superior	0.3158 ± 0.1396 (0.2878 ± 0.1226)	0.3226 ± 0.1527	−0.0068 ± 0.0436 (−0.0348 ± 0.0879)
Inferior	0.3434 ± 0.1783 (0.3367 ± 0.1953)	0.3095 ± 0.1495	0.0339 ± 0.0674 (0.0272 ± 0.0796)

Nonopen-angle eyes			

All quadrants	0.0678 ± 0.0783 (0.0752 ± 0.0694)	0.0681 ± 0.0590	−0.0004 ± 0.0174 (−0.0016 ± 0.0140)
Nasal	0.0813 ± 0.0818 (0.0826 ± 0.0754)	0.0760 ± 0.0717	0.0053 ± 0.0122 (−0.0008 ± 0.0115)
Temporal	0.0772 ± 0.0620 (0.756 ± 0.0599)	0.0781 ± 0.0597	−0.0009 ± 0.0237 (−0.0025 ± 0.0181)
Superior	0.0484 ± 0.0468 (0.0482 ± 0.0435)	0.0491 ± 0.0445	−0.0007 ± 0.0123 (−0.0009 ± 0.0112)
Inferior	0.0640 ± 0.0576 (0.0669 ± 0.0566)	0.0692 ± 0.0570	−0.0051 ± 0.0185 (−0.0022 ± 0.0151)

**Table 6:** Mean (± standard deviation) AOD750 (mm) from each observer and their differences.

	Observer AZC (with adjudicator) (mm)	Observer SR (session 1) (mm)	Difference (with adjudicator) (mm)
All eyes			

All quadrants	0.411 ± 0.421 (0.404 ± 0.417)	0.400 ± 0.401	**0.011 ± 0.067** **(0.004 ± 0.072)**
Nasal	0.447 ± 0.427 (0.434 ± 0.427)	0.433 ± 0.419	0.013 ± 0.064 (0.001 ± 0.056)
Temporal	0.458 ± 0.520 (0.455 ± 0.522)	0.448 ± 0.495	0.010 ± 0.049 (0.008 ± 0.047)
Superior	0.355 ± 0.349 (0.343 ± 0.325)	0.358 ± 0.349	−0.003 ± 0.042 (−0.015 ± 0.074)
Inferior	0.383 ± 0.380 (0.384 ± 0.380)	0.360 ± 0.330	0.023 ± 0.100 (0.024 ± 0.098)

Open-angle eyes			

All quadrants	0.799 ± 0.430 (0.781 ± 0.437)	0.768 ± 0.408	0.031 ± 0.097 (0.013 ± 0.109)
Nasal	0.832 ± 0.439 (0.810 ± 0.456)	0.801 ± 0.446	0.032 ± 0.098 (0.009 ± 0.076)
Temporal	0.920 ± 0.576 (0.923 ± 0.573)	0.901 ± 0.524	0.019 ± 0.073 (0.021 ± 0.071)
Superior	0.706 ± 0.307 (0.671 ± 0.282)	0.708 ± 0.309	−0.002 ± 0.051 (−0.037 ± 0.107)
Inferior	0.735 ± 0.377 (0.723 ± 0.400)	0.662 ± 0.326	0.073 ± 0.140 (0.061 ± 0.152)

Nonopen-angle eyes			

All quadrants	0.166 ± 0.133 (0.166 ± 0.131)	0.167 ± 0.131	−0.001 ± 0.034 (−0.001 ± 0.032)
Nasal	0.203 ± 0.155 (0.197 ± 0.154)	0.201 ± 0.154	0.002 ± 0.023 (−0.004 ± 0.040)
Temporal	0.166 ± 0.127 (0.160 ± 0.128)	0.161 ± 0.130	0.005 ± 0.025 (−0.001 ± 0.021)
Superior	0.134 ± 0.108 (0.137 ± 0.104)	0.138 ± 0.108	−0.004 ± 0.037 (−0.001 ± 0.041)
Inferior	0.160 ± 0.137 (0.170 ± 0.137)	0.169 ± 0.129	−0.009 ± 0.046 (0.001 ± 0.024)

**Table 7:** Interobserver correlation coefficient (ICC) for TISA750 and AOD750.

	TISA750			AOD750		
	By 2 observers (with adjudicator)			By 2 observers (with adjudicator)		
	ICC	|*μ*_d_|/SD_d_	|*μ*_d_|/SD_TISA_	ICC	|*μ*_d_|/SD_d_	|*μ*_d_|/SD_AOD_
All eyes						

All quadrants	0.981 (0.972)	0.098 (0.028)	0.020 (0.007)	0.986 (0.984)	0.029 (0.031)	0.027 (0.011)
Nasal	0.979 (0.987)	0.233 (0.033)	0.047 (0.005)	0.988 (0.992)	0.087 (0.100)	0.032 (0.002)
Temporal	0.993 (0.996)	0.050 (0.045)	0.006 (0.004)	0.995 (0.996)	0.200 (0.048)	0.020 (0.015)
Superior	0.986 (0.933)	0.110 (0.250)	0.019 (0.093)	0.993 (0.975)	0.108 (0.024)	0.008 (0.045)
Inferior	0.960 (0.954)	0.211 (0.178)	0.060 (0.055)	0.959 (0.961)	0.196 (0.042)	0.066 (0.068)

Open-angle eyes						

All quadrants	0.957 (0.936)	0.183 (0.009)	0.054 (0.003)	0.971 (0.967)	0.320 (0.119)	0.074 (0.032)
Nasal	0.955 (0.973)	0.236 (0.082)	0.073 (0.020)	0.975 (0.987)	0.327 (0.118)	0.073 (0.021)
Temporal	0.992 (0.995)	0.070 (0.065)	0.009 (0.020)	0.991 (0.992)	0.260 (0.296)	0.035 (0.039)
Superior	0.958 (0.785)	0.156 (0.396)	0.048 (0.282)	0.987 (0.932)	0.039 (0.346)	0.006 (0.131)
Inferior	0.902 (0.892)	0.503 (0.342)	0.215 (0.165)	0.907 (0.908)	0.521 (0.401)	0.216 (0.174)

Nonopen-angle eyes						

All quadrants	0.959 (0.971)	0.023 (0.114)	0.006 (0.028)	0.968 (0.970)	0.029 (0.031)	0.011 (0.011)
Nasal	0.985 (0.987)	0.434 (0.070)	0.071 (0.012)	0.989 (0.967)	0.087 (0.100)	0.010 (0.028)
Temporal	0.928 (0.956)	0.038 (0.138)	0.015 (0.042)	0.982 (0.987)	0.200 (0.048)	0.039 (0.010)
Superior	0.966 (0.969)	0.057 (0.080)	0.016 (0.021)	0.943 (0.929)	0.108 (0.024)	0.035 (0.009)
Inferior	0.947 (0.966)	0.276 (0.146)	0.092 (0.040)	0.943 (0.984)	0.196 (0.042)	0.068 (0.005)

**Table 8:** Post hoc analysis of correlation of quadrants within an eye.

Variable	Method	*r*
SSX by SR from session 1	Mixed-effect model	0.075
SSY by SR from session 1	Mixed-effect model	−0.051
Distance of SSL between SR and AZC	Mixed-effect model	0.078
Agreement within 80 um between SR and AZC	GEE with repeated measure and binomial link	0.016
TISA by SR from session 1	Mixed-effect model	0.911
AOD by SR from session 1	Mixed-effect model	0.906
