# Supplementary figures and images for: Reproducibility of Scleral Spur Identification and Angle Measurements Using Fourier Domain Anterior Segment Optical Coherence Tomography
Source: J Ophthalmol. 2012 Nov 1;2012:487309. doi: 10.1155/2012/487309 (PMC3503366; doi:10.1155/2012/487309)

**Supplementary Figure 1**

**
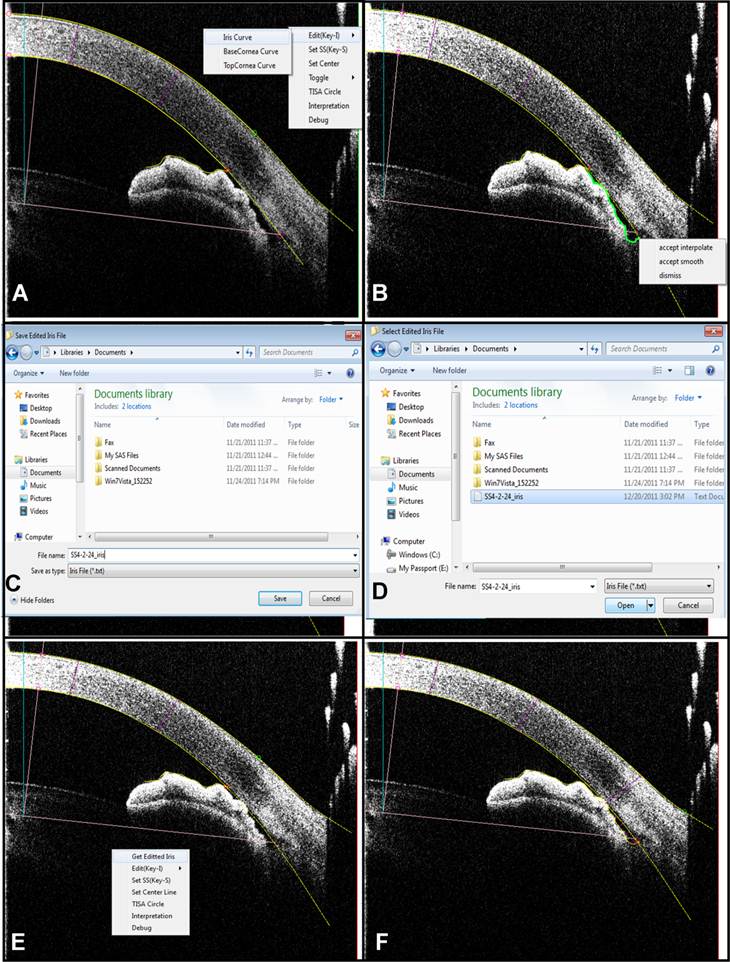
**

Supplement: Supplementary file 1 — Illustrations are Edit and Save Functions in Anterior Chamber Analysis and Interpretation (ACAI) software. After manually identifying the scleral spur landmark (SSL [SS in the figure]), click on the edge detection button (not shown), and the anterior and posterior corneal edges as well as the anterior iris are automatically detected (A, yellow lines). In some eyes, especially nonopen angle eyes, iris surface detection may need manual adjustment (B, green line). Trabecular Iris Surface Area at 750 μm (TISA750) and Angle Opening Distance at 750 μm (AOD750) can be calculated based on the identified SSL and the edited edges (not shown). The edited iris can then be saved to a file (C). To compare TISA750 and AOD750 calculations obtained from the other set of SSL identifications on the same image, the saved iris file would be loaded first (D). After the SSL is identified and the edges automatically detected (by the yellow lines, as in A), the user then clicks on “Get Edit Iris” (E). The iris surface edge is from the previous saved iris file (F). The impact of SSL on TISA750 and AOD750 can then be examined with the same manually edited iris edges. [file 487309.f1.docx]
